# PEGylated Liposomes Loaded with Carbamate Inhibitor ANP0903 Trigger Apoptosis by Enhancing ER Stress in HepG2 Cancer Cells

**DOI:** 10.3390/ijms24054552

**Published:** 2023-02-25

**Authors:** Carla Caddeo, Rocchina Miglionico, Roberta Rinaldi, Ilaria Nigro, Daniela Lamorte, Lucia Chiummiento, Paolo Lupattelli, Maria Funicello, Rosarita D’Orsi, Donatella Valenti, Valentina Santoro, Anna Maria Fadda, Faustino Bisaccia, Antonio Vassallo, Maria Francesca Armentano

**Affiliations:** 1 Department of Scienze della Vita e dell’Ambiente, Sezione di Scienze del Farmaco, University of Cagliari, Via Ospedale 72, 09124 Cagliari, Italy; 2 Department of Scienze, University of Basilicata, Viale dell’Ateneo Lucano 10, 85100 Potenza, Italy; 3 Laboratory of Preclinical and Translational Research, Centro di Riferimento Oncologico della Basilicata (IRCCS-CROB), 85028 Rionero in Vulture, Italy; 4 Department of Chimica, Sapienza University of Roma, p.le Aldo Moro 5, 00185 Roma, Italy; 5 Department of Farmacia, University of Salerno, Via Giovanni Paolo II 132, 84084 Salerno, Italy; 6 Spinoff TNcKILLERS s.r.l., Viale dell’Ateneo Lucano 10, 85100 Potenza, Italy

**Keywords:** carbamate inhibitor, liposomes, targeted therapy, hepatic cancer cells, ER stress, apoptosis, proteasome inhibition

## Abstract

Liver cancer is one of the most common causes of cancer death worldwide. In recent years, substantial progress has been made in the development of systemic therapies, but there is still the need for new drugs and technologies that can increase the survival and quality of life of patients. The present investigation reports the development of a liposomal formulation of a carbamate molecule, reported as ANP0903, previously tested as an inhibitor of HIV-1 protease and now evaluated for its ability to induce cytotoxicity in hepatocellular carcinoma cell lines. PEGylated liposomes were prepared and characterized. Small, oligolamellar vesicles were produced, as demonstrated by light scattering results and TEM images. The physical stability of the vesicles in biological fluids was demonstrated in vitro, alongside the stability during storage. An enhanced cellular uptake was verified in HepG2 cells treated with liposomal ANP0903, resulting in a greater cytotoxicity. Several biological assays were performed to elucidate the molecular mechanisms explaining the proapoptotic effect of ANP0903. Our results allow us to hypothesize that the cytotoxic action in tumor cells is probably due to the inhibition of the proteasome, resulting in an increase in the amount of ubiquitinated proteins within the cells, which in turn triggers activation of autophagy and apoptosis processes, resulting in cell death. The proposed liposomal formulation represents a promising approach to deliver a novel antitumor agent to cancer cells and enhance its activity.

## 1. Introduction

**Figure 1:**
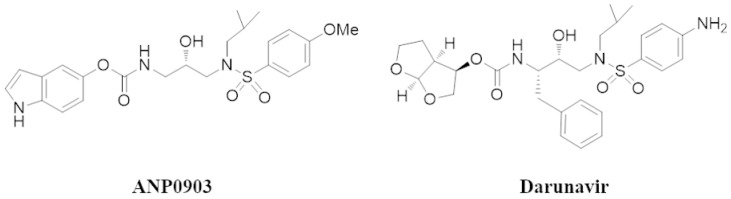
Chemical structures of ANP0903 and darunavir.

According to the World Health Organization, cancer is a leading cause of death worldwide, accounting for nearly 10 million deaths in 2020. Liver cancer is one of the most common malignant tumors, and hepatocellular carcinoma (HCC) is the predominant, common pathological type of liver cancer [1]. Multiple conventional approaches are being used in treating hepatocellular carcinoma, including liver resection, liver transplantation, local ablative therapy, transarterial therapy, and systemic regular drug therapies (mainly tyrosine kinase inhibitors) [2,3]. In this regard, issues related to drug resistance, both intrinsic and acquired, toxicity, low drug bioavailability, and side effects limit current treatment methods and undermine positive clinical outcomes [4]. Therefore, there is an urgent need to identify new options for a systemic therapy capable of overcoming these critical issues and being more effective. A strategy to accelerate the otherwise considerable time required to identify new therapeutic targets and reduce costs for pharmaceutical companies can be the repositioning of already approved drugs, whose pharmacokinetics and toxicity data are already known. The potential repurposing of the human immunodeficiency virus protease inhibitors (HIV-PIs) as antitumoral chemotherapeutics (mainly nelfinavir, saquinavir, and ritonavir) comes from the early observation of their effects on HIV-related Kaposi’s sarcoma [5]. However, numerous preclinical studies [6] suggesting direct off-target anticancer effects, sometimes also surmounting the drug resistance issue [7], were reported. Given these pleiotropic effects, in this study we wanted to ascertain the potential antitumor activity of ANP0903, a carbamate molecule considered as an analog of commercial HIV-PI darunavir (Figure 1), whose inhibitory capacity against HIV-1 protease was previously evaluated (indicated as compound 10c in [8]).

Furthermore, with the aim of improving the bioavailability and bioactivity of ANP0903, we considered that one approach to increase the pharmacological treatment efficacy could be represented by the use of nanoparticles, which were also proposed in the HCC management [9,10]. Liposomes, vesicles composed of one or more phospholipid bilayers, represent a promising carrier for drug delivery, with many liposome-based drug formulations commercially available. They are among the most commonly used carriers in the targeted treatment of cancers, with some examples proposed for the treatment of HCC [11,12]. Some of the shortcomings of liposomes, such as uptake by immune cells, can be overcome by modifying their surface with polymers, such as polyethyleneglycol (PEG), which is a flexible, non-fouling, hydrophilic polymer used to passivate nanoparticles to extend the half-life in blood circulation [13]. Furthermore, to increase the selectivity of liposomes for disease targets and control the delivery timing or dosage, several engineering strategies have been adopted. The surface of liposomes has been functionalized with specific ligands in order to allow targeted delivery and further enhance the efficacy of the incorporated drug [14]. In this study, PEG-liposomes were prepared to load and deliver ANP0903 to hepatocarcinoma cells. The liposomes were characterized to evaluate the main physico-chemical and technological properties, such as size, surface charge, morphology, storage stability, and stability in biological fluids. A preliminary cytotoxicity screening was performed on four liver cell lines treated with the free ANP0903. Since a higher cytotoxicity was found towards HepG2 cells, the latter were chosen for further investigation. The efficacy of the liposomal drug in comparison with the free drug was assessed by carrying out a panel of experiments to characterize the dysregulated molecular mechanisms.

## 2. Results

### 2.1. Vesicle Production and Characterization

The present study aimed to develop a nanoformulation for a synthetic antitumor drug, ANP0903, to the hepatic cancer cell line HepG2. PEGylated liposomes were produced, characterized, and tested in vitro to assess the potential enhancement of the antitumor activity of the synthesized carbamate inhibitor. In parallel to ANP0903 PEG-liposomes, empty PEG-liposomes and non-PEGylated liposomes were prepared to assess the effect of the drug and the PEGylation, respectively. The light scattering results, as reported in Table 1, showed that the empty PEG-liposomes were approximately 87 nm in diameter, monodispersed (PI 0.24), and negatively charged (ZP −19 mV).

**Figure 2:**
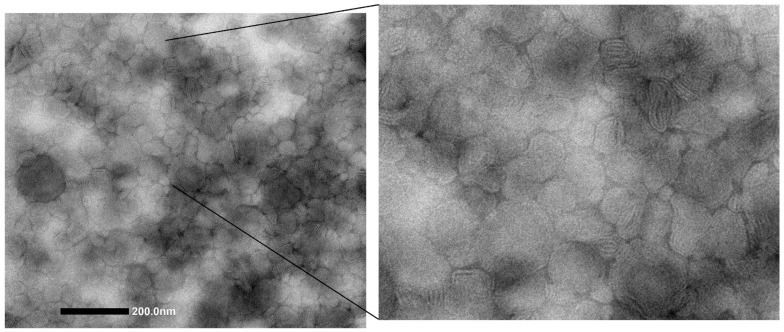
TEM image of ANP0903 PEG-liposomes.

The loading of ANP0903 did not affect the size of the vesicles, nor the PI and the zeta potential (Table 1). The effect of the PEGylation on the vesicles was evaluated by preparing non-PEGylated liposomes, both empty and loaded with ANP0903. As shown by the results reported in Table 1, the non-PEGylated liposomes, irrespective of the presence of the drug, were slightly smaller than PEG-liposomes, but not in a statistically significant manner. On the contrary, the effect of the PEGylation on the zeta potential was statistically significant. The values were less negative, which suggests that the PEG-phospholipid is arranged in a way that exposes the positively charged amine groups. The entrapment efficiency of the PEG-liposomes was high (78% ± 7.8), and the amount of the loaded ANP0903 did not diminish significantly after a two-month storage period (75% ± 5.7; *p* > 0.05). TEM observation of ANP0903 PEG-liposomes confirmed the formation of the vesicles, which were characterized by irregularly spherical shape, oligolamellarity, and small size (Figure 2), in alignment with the light scattering data.

The PEG-liposomes were monitored during storage by evaluating the mean diameter, polydispersity, and zeta potential. After 2 months of storage, no signs of significant physical alteration were detected, as demonstrated by the fact that the three parameters changed only slightly (MD 96 nm ± 4.7, PI 0.28 ± 0.02, ZP −16 mV ± 6.4). In addition, the stability of the PEG-liposomes was evaluated in a simulated biological fluid. Hanks’ solution is commonly used to simulate body fluids, as it contains inorganic salts and glucose that confer a similar osmotic pressure as that of intercellular fluids [15]. PEG-liposomes remained essentially unaltered after dilution and incubation for 24 h with Hank’s solution. The mean diameter and PI did not change significantly from the initial values, being around 80 nm and 0.20, respectively (Table 2). This indicates that their structure was preserved despite osmotic stress caused by the high ions’ concentration in the medium. On the other hand, the zeta potential values varied, approaching neutrality (Table 2), due to the ions adsorbed on the vesicles’ surface.

### 2.2. ANP0903 Affected Viability of Hepatoma Cell Lines in a Dose-Dependent Manner

**Figure 3:**
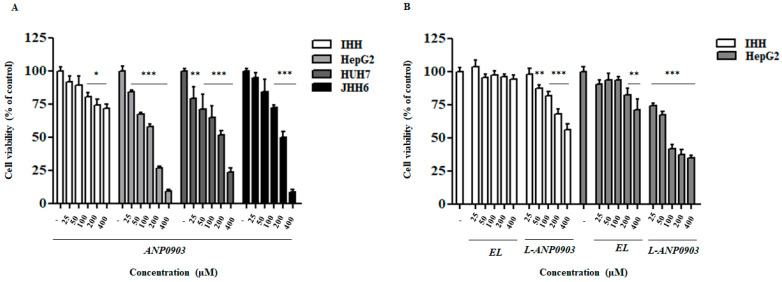
Effect of ANP0903 and its liposomal formulation on cell viability. MTT assays were performed (**A**) on normal hepatocytes (IHH) and on three different hepatoma cell lines (HepG2, HUH7, and JHH6) treated for 24 h with ANP0903 at indicated concentrations (25–400 µM) and (**B**) on IHH and on HepG2 cells treated for 24 h with ANP0903 PEG-liposomes (L-ANP0903) at indicated concentrations (25–400 µM) or empty PEG-liposomes (EL), which were subjected to the same dilutions as ANP0903 and L-ANP0903 to reach the above concentrations. Cell viability was expressed as a percentage relative to control cells treated with vehicle (indicated with minus sign or with CTRL abbreviation). All data are expressed as means ± Standard Error (SE) of three experiments, each performed in triplicate, and statistical significance was evaluated by one-way ANOVA followed by Dunnett’s post hoc test (* *p* < 0.05, ** *p* < 0.01, *** *p* < 0.001 vs. CTRL).

To evaluate the putative cytotoxic effects of ANP0903, three hepatocarcinoma cell lines (HepG2, HuH7, and JHH6, with different degrees of differentiation) and a healthy liver cell line (IHH) were exposed to several concentrations (25, 50, 100, 200, and 400 µM) of free ANP0903 for 24 h, and cell viability was estimated by the MTT assay. As shown in Figure 3A, the cytotoxicity was found significantly greater in the three hepatocellular carcinoma cell lines than in the non-tumor cells. Moreover, since a lower IC_50_ value was measured in HepG2 cells, the latter were chosen for further experiments. In addition, L-ANP0903 showed cytotoxic effects higher toward the tumoral cell line (HepG2) than the healthy liver cell line (IHH), and an efficacy superior to the free molecule (IC_50_ 102.5 vs. 150.1 μM; Table 3). Empty PEG-liposomes (EL) showed very low toxicities in both cell lines (Figure 3B and Table 3). The Shapiro–Wilk test was used to assess the normal distribution of the acquired data.

### 2.3. ANP0903 Induced Morphological Alterations in HepG2-Treated Cells

**Figure 4:**
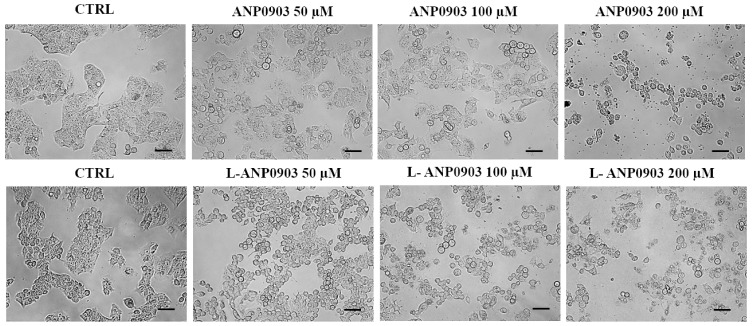
Morphological analysis of cells after exposure to ANP0903 and its liposomal formulation. HepG2 cells were observed under light microscopy with 40× magnification after exposure to the indicated concentrations for 24 h. Scale bars: 100 μm.

HepG2 cell cultures incubated with free or liposomal ANP0903 (50, 100, and 200 µM) were photographed by using a contrast phase microscopy, revealing detachment, rounded morphology, and a dose-dependent vacuolization (Figure 4).

### 2.4. Treatment of ANP0903 Significantly Induced Apoptosis in Hepatoma Cells

**Figure 5:**
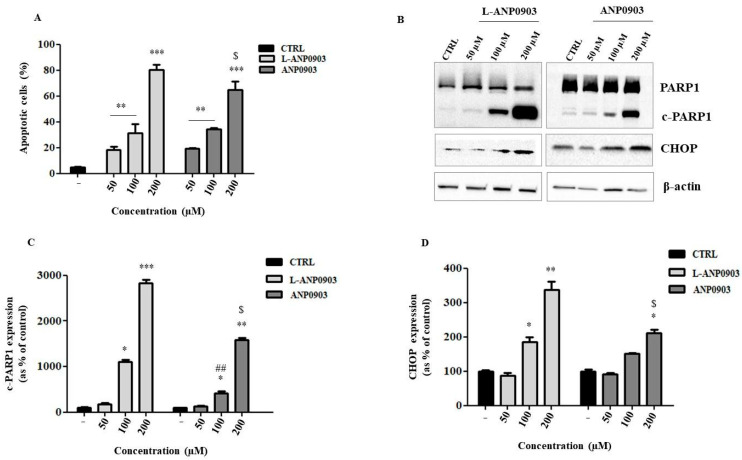
Apoptotic activity of ANP0903 and its liposomal formulation in HepG2 cells after 24 h of treatment. (**A**) FACS analysis. HepG2 cells treated with vehicle were used as negative control (CTRL). All data are expressed as means ± Standard Error (SE) of three experiments, each performed in triplicate, and statistical significance was evaluated by one-way ANOVA followed by Dunnett’s post hoc test or unpaired *t*-test (**B**). Representative Western blot. (**C**,**D**) Densitometric analysis of PARP1 cleavage and CHOP protein expression. Protein levels were presented as the fold change relative to control cells treated with vehicle (CTRL). β-actin was used as the loading control. Data are expressed as means ± Standard Error (SE) of three independent experiments. Kruskal–Wallis or Mann–Whitney nonparametric tests were performed; * *p* < 0.05, ** *p* < 0.01, *** *p* < 0.001 all samples vs. CTRL; ^##^ *p* < 0.01 ANP0903 100 µM vs. L-ANP0903 100 µM and ^$^ *p* < 0.05.

Exposure of HepG2 cells to both free and formulated ANP0903 (50, 100, and 200 µM) for 24 h elevated the number of apoptotic cells, as shown in Figure 5A after staining with annexin V-FITC and PI.

The treatment of the cells with ANP0903 PEG-liposomes was more effective, and this is indicated by a significant increase in the percentage of cells in apoptosis in the liposomal formulation compared with that treated with free ANP0903 (Figure 5A). Shapiro–Wilk test was used to assess the normal distribution of the acquired data. The induction of apoptosis was further evaluated by monitoring the expression of the cleaved form of the protein poly(ADP-ribose)polymerase (PARP-1), a known substrate of activated caspase 3, and of the transcription factor CHOP, whose overexpression is induced by a ER stress condition and allows for the transcription of proapoptotic genes. In both cases, the induction of apoptosis occurs, but the treatment of HepG2 cells with ANP0903 is more effective using its liposomal formulation (Figure 5B–D).

### 2.5. Intracellular Intake of ANP0903

**Figure 6:**
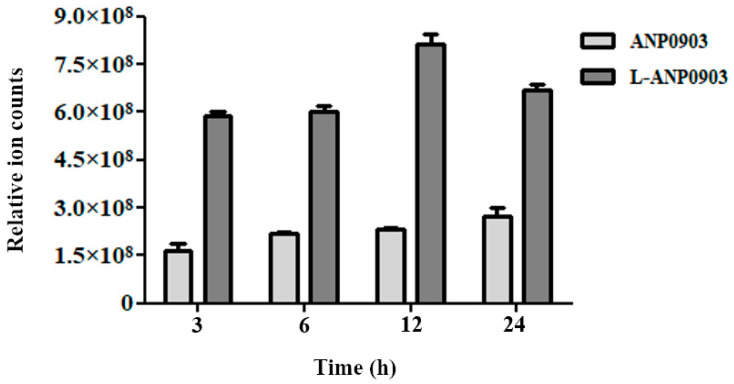
ANP0903 and liposomal ANP0903 intracellular intake. HepG2 cells were treated with 100 µM ANP0903, as free molecule in solution or formulated in PEG-liposomes, at indicated times and the amount of ANP0903 accumulated in HepG2 cells was reported as an area of the chromatographic peak. Results were reported as means of three independent replicates ± Standard Deviation (SD).

In order to evaluate the ANP0903 cell intake after cell incubation with the free compound or with its liposomal formulation, LC–MS analyses were performed. To obtain a good resolution and sensitivity for the ANP0903 detection, different chromatographic conditions, in terms of eluent composition and pH, were tested. LC–MS analyses can provide information on the amount of ANP0903 within cells upon different treatments. HepG2 cells were incubated with ANP0903 in solution or in PEG-liposomes (100 µM) up to 24 h. The results confirmed that the use of the liposomal formulation clearly increased the amount of intracellular ANP0903, at different tested times. The amount of ANP0903 detected within the cells at 24 h was higher (~2.5-fold) when it was formulated in liposomes than in solution (Figure 6).

### 2.6. Liposomal ANP0903 Induced ER Stress and Stimulated Autophagic Mechanisms

**Figure 7:**
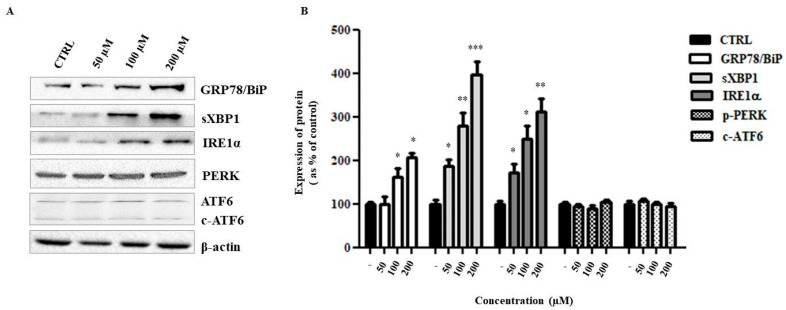
Activation of IRE1-XBP1 branch of UPR in HepG2 cells treated with liposomal ANP0903 for 24 h at indicated concentrations. (**A**) Representative Western blot and (**B**) densitometric analysis of immunoreactive bands. Protein levels were presented as the fold change relative to control cells (CTRL). PERK and ATF6 branches were not activated, as highlighted by unaltered expressions of the proper markers. β-actin was used as the loading control. Data are expressed as means ± Standard Error (SE) of three independent experiments, and statistical significance was evaluated by Kruskal–Wallis test (* *p* < 0.05, ** *p* < 0.01, *** *p* < 0.001 vs. CTRL).

Literature data report that several HIV-PIs and their analogs have anticancer activity via induction of ER stress (ERS) and autophagy [16,17,18,19]. Therefore, in this study, it was verified whether the exposure of HepG2 cells to ANP0903 PEG-liposomes, which showed to be more effective, triggered unfolded protein response (UPR) and autophagy mechanisms. As shown in Figure 7A,B, the protein expression of the main ER stress marker, namely the GRP78/BiP chaperone, was significantly altered, and the IRE1α branch of the UPR seemed to be activated (PERK and ATF6 branches did not highlight activation, Figure 7).

**Figure 8:**
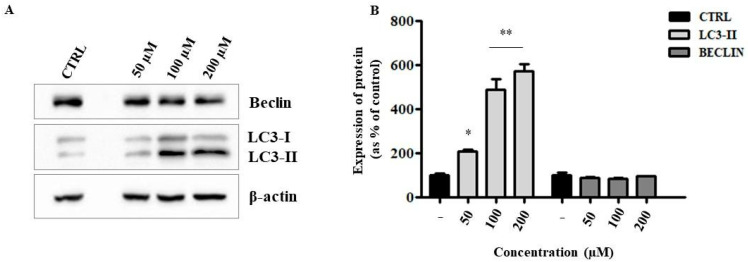
Evaluation of expression levels of autophagy-related proteins. Representative Western blot images (**A**) and relative densitometric analyses (**B**) performed on the lysates of HepG2 cells treated with the indicated concentrations of liposomal ANP0903. β-actin was used as a loading control. Protein levels were presented as the fold change relative to control cells (CTRL). Data are expressed as means ± Standard Error (SE) of three independent experiments and statistical significance was evaluated with Kruskal–Wallis test (* *p* < 0.05, ** *p* < 0.01 vs. CTRL).

Cellular ER stress could trigger autophagic processes [20], therefore we evaluated the protein expression of two important markers of this mechanism, namely, the microtubule-associated protein 1A⁄1B-light chain 3 (LC3), which exists as a cytoplasmic form (LC3-I) or as a membranous form (LC3-II), which is a component of autophagosomes, and the protein Beclin-1, an important regulator of early-stage autophagy. Our results, which show an increase in LC3-II expression and, conversely, an unaltered Beclin-1 expression value (Figure 8), suggest an independent mTOR activation of autophagy. Both evaluations support the hypothesis of caspase-independent cell death following treatment with the liposomal formulation.

### 2.7. Could Liposomal ANP0903 Inhibit the Proteasome?

**Figure 9:**
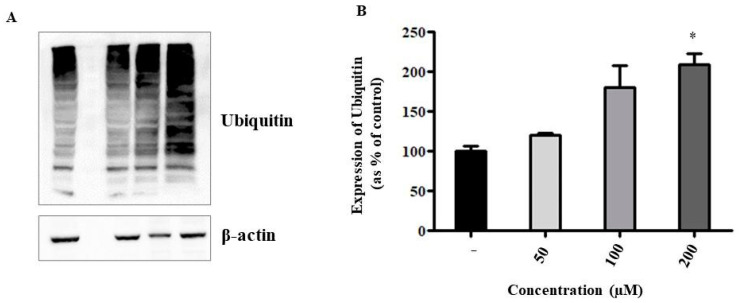
Protein ubiquitination assessment in HepG2 cells exposed to liposomal ANP0903. (**A**) Representative Western blot and (**B**) densitometric analysis of immunoreactive bands. Protein levels were presented as the fold change relative to control cells (CTRL). β-actin was used as the loading control. Data are expressed as means ± Standard Error (SE) of three independent experiments and statistical significance was evaluated with Kruskal-Wallis test (* *p* < 0.05 vs. CTRL).

Several studies report the proteasomal complex, a fundamental mechanism of protein degradation, as an unusual intracellular target of HIV-PIs and/or their analogues [21,22]. Its inhibition therefore determines an overload of proteins that is not sustainable by the cell, which therefore dies. This pleiotropic effect can be investigated through an indirect analysis of the inhibition of the proteasome, which is through the evaluation of the levels of intracellular ubiquitination. Our results highlighted a dose-dependent increase in the levels of ubiquitinated proteins within the cells (Figure 9) exposed to treatment with ANP0903 PEG-liposomes, confirming our initial hypothesis.

## 3. Discussion

Hepatocellular carcinoma (HCC) is the most common type of liver cancer, accounting for 80% of all cases, making it the most relevant to focus on [23]. The commonly established methods for the diagnosis of HCC are imaging techniques (ultrasound, computed tomography, or magnetic resonance imaging) and the quantification of circulating overexpressed biomarkers, such as alphafetoprotein (AFP) [2]. Multiple approaches are currently available for the treatment of HCC, depending on the tumor stage and underlying conditions [4]. Surgical resection and transplant are the first-line treatments. The second-line options include locoregional treatments (ablation, embolization, and radiation) and systemic drug therapies. The latter include immunotherapy, which involves the use of immune checkpoint inhibitors; chemotherapy, which involves the use of traditional drugs, such as gemcitabine, cisplatin, and 5-fluorouracil, that are often more effective in destroying cancer cells if combined; and targeted therapy, which involves the use of kinase inhibitors that block tyrosine kinases and thus stop the growth of the cancer, or monoclonal antibodies that target vascular endothelial growth factor, affecting a tumor’s ability to form new blood vessels and thus to grow [24].

Although significant progress has been made in early diagnosis and treatment of liver cancer over the last two decades, the prognosis remains poor [2]. There are multiple reasons that can explain these insufficient outcomes, which are low drug bioavailability and non-specificity, and which can determine the high risk of side effects with low drug concentration in the target tissue. The development of new systemic drug therapies is a slow process, so there is the growing need for new effective treatment options to be developed in shorter times that can improve the survival and quality of life of patients [3].

HIV-1 protease inhibitors (HIV-PIs) are peptidomimetic drugs that contain an analogue of the phenylalanine-proline sequence targeted by the HIV-1 aspartyl protease [19]. Several lines of evidence suggest that, in addition to the antiretroviral properties, HIV-PIs exert pleiotropic pharmacological effects, including anticancer activities [5]. Apart from a direct anti-proliferative effect, HIV-PIs have also been shown to inhibit the growth of a variety of cancers by blocking angiogenesis [25], affecting the cell cycle [26], and promoting apoptosis and ER stress [19] and autophagy and proteasome inhibition [22]. Moreover, several reports have shown that HIV-PIs can be used as chemo- and radio-sensitizers [27,28].

The recognition of HIV-PIs as potential anticancer agents has intensified the effort to understand their mechanism of action and, if possible, to develop more potent and effective derivatives with wider availability and less toxicity than the anticancer drugs in use, which can improve the survival and quality of life of patients. In this scenario, nanotechnologies can contribute to expanding the armamentarium against tumors. Both inorganic and organic nanoparticles have been proposed for cancer therapy [29]. Among organic nanoparticles, liposomes are the very first nanosystem approved for the treatment of cancer, and still are the best-investigated platform in clinical trials and academic research [30,31,32,33]. They offer numerous advantages over therapies with free drugs: increased solubility and bioavailability of the loaded drug, controlled/constant rate of drug released over the desired timescale, protection of the loaded drug, selectivity against cancer targets, thus increased efficacy, and reduced dose of the administered drug, thus lower systemic toxicity [34,35,36]. For liposomes to be an efficient carrier system, they need to be properly formulated by tailoring their characteristics as a function of the drug and the therapeutic purpose. In this study, we developed a liposomal formulation by a simple and rapid procedure that involves the dispersion of the components in aqueous medium and the sonication to allow the formation of vesicles in the nanoscale range. Since the formulation was intended for parenteral administration, the liposomes were prepared with PEGylated phospholipids to prolong circulation time in the blood stream. PEGylated liposomes were used for loading and administering a synthetic drug, in this study called ANP0903, a carbamate molecule previously investigated (indicated as compound 10c in [8]) as an HIV-1 protease inhibitor. We wanted to ascertain the potential antitumor activity of ANP0903, due to the pleiotropic effects demonstrated by many HIV-PIs, comparing the biological effects of both the free and the nanoformulated molecule. We preliminarily tested the putative cytotoxic effects of the ANP0903 free molecule towards three pathological liver cell lines, HepG2, HuH7, and JHH6, characterized by high, medium, and low hepatocytic differentiation, respectively, compared to an immortalized non-pathological liver cell line IHH. Our results showed a lower cytotoxicity in the healthy cell line and similar cytotoxic effects in the pathological cell lines, with a slightly higher IC_50_ in HepG2 cells. Furthermore, we chose the HepG2 cell line for the subsequent assays, considering the characterization of the dysregulated molecular mechanisms carried out on a more differentiated tumor line more effective. Liposomal ANP0903 exhibited a slightly greater efficacy (i.e., lower IC_50_) compared to the free molecule. With both treatments, a dose-dependent mechanism of death by apoptosis was triggered, as evidenced by the FACS analysis. However, the analysis of apoptosis markers, such as c-PARP and CHOP, justified, in subsequent assays, the use of only liposomal ANP0903, which proved to be more effective. The explanation of this greater cytotoxicity could lie in the more efficient internalization of PEG-liposomes by the cells. As of yet, the mechanism of liposome–cell interaction is not fully elucidated, and more than one may occur at the same time. Endocytosis is regarded as the primary mechanism of entry, but micropinocytosis and membrane fusion can also be involved. Our results showed that the incorporation of ANP0903 into PEG-liposomes facilitated the transport of the molecule across the cell membrane and its accumulation into the cytoplasm, as demonstrated by a double amount of ANP0903 detected within the cells after the exposure to liposomal ANP0903 in comparison with free ANP0903 (Figure 6).

It is known that several HIV-PIs (or their analogues) exert their cytotoxic activity through the induction of ER stress, sometimes triggered by proteasome inhibition [17,22], justifying their use as possible anticancer agents [37]. Therefore, we wanted to verify this possibility by treating the HepG2 cells with different concentrations of liposomal ANP0903 and assessing, by Western blotting, the levels of expression of some markers connected to these processes. Our results suggest that liposomal ANP0903 was able to activate the IRE1α branch of the UPR (as confirmed by the upregulation also of sXBP1) because of a strong ER stress state, proven by the increased expression level of the representative ER-resident chaperone GRP78/BiP (Figure 7). The other two branches of the UPR (ATF6 and PERK) were not significantly activated. sXBP1 is responsible for the expression of chaperones and genes involved in the ERAD system, which is capable of providing the disposal of unfolded and misfolded proteins from the ER. The level of protein ubiquitination is considered as a surrogate marker of proteasome inhibition [38]: given the high amount of ubiquitinated proteins in the cells after treatment with liposomal ANP0903 (Figure 9), we hypothesized a direct or indirect inhibition of the proteasome that will justify further investigations to better understand its mechanism of action.

Liposomal ANP0903 was found to hinder the regular disposal of the proteins coming from the ER and thus determining a proteotoxic state responsible for ER stress. Finally, several studies reported that the use of proteasome inhibitors, routinely used in clinical settings, determines an increased expression level of LC3-II, a classic marker of autophagosome formation, as a cellular attempt to reduce stress and restore normal proteostasis. We detected an increased level of LC3-II expression (Figure 8), which further suggests a possible inhibition of the proteasome as a mechanism of action of liposomal ANP0903.

Overall, these results make plausible the idea that this molecule can determine an inhibition of the proteasome and that the accumulated unfolded proteins eventually result in ER stress, also triggering the autophagic process in the cells. Moreover, they point to the fact that the prepared PEG-liposomes could efficiently carry ANP0903 and potentiate its antitumor activity in hepatocarcinoma cells. This confirms previous findings concluding that lipid-based nanoparticles hold great promise in improving the treatment outcomes of antitumor agents for HCC. Indeed, liposomes were demonstrated to significantly enhance the cytotoxic activity of conventional chemotherapeutic agents (e.g., docetaxel, 5-fluorouracil, and doxorubicin) [39,40,41] or phytochemicals (e.g., catechin and resveratrol) [42,43], alone or in combination with microwave ablation or radiotherapy [44,45]. Nevertheless, despite the intensive research efforts, clinically significant results have not been achieved yet, and further improvements are required. Mahmoud et al. recently highlighted that a focus should be put on the methods of preparation of the nanoparticle delivery systems in order to increase bench to bedside translation [9]. They suggested that novel, green, and ecofriendly methods that do not depend on organic solvents would improve the scalability of production and decrease production costs, while eliminating the risk of the presence of toxic residuals. This perfectly aligns with the formulation approach adopted in our investigation, which was based on an easy, organic, solvent-free, and efficient procedure leading to nanosized, stable, biocompatible vesicles.

## 4. Materials and Methods

### 4.1. Materials

1,2-Dipalmitoyl-sn-glycero-3-phosphocholine (DPPC), Phospholipon90G (>90% phosphatidylcholine; P90G), and [N-(Carbonyl-methoxypolyethylene glycol-5000)-1,2-dipalmitoyl-sn-glycero-3-phosphoethanolamine, sodium salt] (MPEG-5000-DPPE) were purchased from Lipoid GmbH (Ludwigshafen, Germany). Ethanol 96% was purchased from Merck (Milan, Italy). ANP0903 (Figure 1) (1H-indol-5-yl(2-hydroxy-3-(N-isobutyl-4-methoxyphenylsulfonamido) propyl) carbamate) was synthesized as previously reported [8]. Dulbecco’s Modification of Eagle’s Medium was purchased from Corning (Corning, New York, NY, USA). Dulbecco’s Modified Eagle’s Medium/Nutrient Mixture F-12 Ham, William’s E medium Dimethyl sulfoxide (DMSO), Trypsin–EDTA Solution, Thiazolyl Blue Tetrazolium Bromide (MTT), and Bradford Reagent were purchased from Sigma Aldrich-Merck (Saint Louis, MO, USA). Dulbecco’s Phosphate Buffered Saline, L–Glutamine, Penicillin-Streptomycin Solution, and Fetal Bovine Serum were purchased from EuroClone (Milan, Italy). Primary antibodies specific for poly-ADP-ribose polymerase (PARP)(#9542, 116/89 kDa), LC3A/B (#12741, 16/14 kDa), CHOP (#2895, 27 kDa), IRE1α (#3294, 130 kDa), Ubiquitin (#3936), Actin (#3700, 45 kDa), Antimouse IgG-HRP-linked (#7076), and Anti-rabbit IgG-HRP-linked (#7074) were purchased from Cell Signaling Technology (CST, Danvers, MA, USA). Primary antibodies specific for Beclin-1 (#849701, 52 kDa), sXBP1 (#647501, 55 kDa), and ATF6 (#853101, 80 kDa) were purchased from Biolegend (San Diego, CA, USA). Primary antibodies specific for GRP78⁄ BiP (#ab21685, 75 kDa) and the anti-rat IgG-HRP-linked (#ab97057) were from Abcam (Cambridge, UK). The primary antibody specific for p-PERK (#SAB5700521, 125 kDa) was purchased from Sigma Aldrich-Merck. The annexin V-PI apoptosis detection kit was purchased from BD Biosciences (San Jose, CA, USA). Methanol, acetonitrile, formic acid, and water for liquid chromatography mass spectrometry (LC-MS) were purchased from Romil Ltd. Pure Chemistry (Cambridge, UK).

### 4.2. Vesicle Preparation and Characterization

To produce ANP0903 PEG-liposomes, in a glass vial, ANP0903 was dissolved in ethanol and then DPPC, P90G, and MPEG-5000-DPPE (lipid molar ratio 51.3:48:0.7) were added, followed by water (Table 3). The dispersion was sonicated (10 cycles, 5 s on and 2 s off) with a Soniprep 150 disintegrator (MSE Crowley, London, UK).

For an appropriate comparison, empty PEG-liposomes were prepared following the above procedure, but without the addition of ANP0903 (Table 3).

Non-PEGylated liposomes (i.e., liposomes without MPEG-5000-DPPE) were also prepared to evaluate the effect of PEGylation on the vesicles’ features (Table 4).

The formation and morphology of the vesicles were examined using transmission electron microscopy (TEM). For the analysis, a drop of the vesicle dispersion was adsorbed on a carbon 200 mesh 3 mm copper grid, stained with 1% phosphotungstic acid, and observed under a JEM 1400-PLUS microscope (Jeol Europe, Croissy-sur-Seine, France) operating at an accelerating voltage of 80 kV.

The average diameter, polydispersity index (PI, a measure of the width of size distribution), and zeta potential of the vesicles were determined via dynamic and electrophoretic light scattering using a Zetasizer nano-ZS (Malvern Panalytical, Worcestershire, UK). For the measurements, samples (*n* > 10) were diluted with water (1:100) and analyzed at 25 °C.

The vesicle dispersions were purified from the non-incorporated ANP0903 by dialysis. Each sample (1 mL) was loaded into Spectra/Por^®^ tubing (12–14 kDa MW cut-off; Spectrum Laboratories Inc., Breda, The Netherlands) and dialyzed against water (2 L) to allow the removal of the non-incorporated drug. After 2 h, both unpurified and purified vesicles were disrupted with methanol, ANP0903 content was determined by high-performance liquid chromatography (Alliance 2690, Waters, Milan, Italy), and the entrapment efficiency (E) was calculated as the percentage of the amount of ANP0903 detected in purified vs. unpurified samples. ANP0903 was detected at 242 nm using an XSelect C18 column (3.5 µm, 4.6 × 100 mm, Waters), and a mobile phase consisting of acetonitrile, water, and acetic acid (99.5:0.48:0.02, *v*/*v*/*v*) delivered at a flow rate of 0.5 mL/min.

### 4.3. Stability of the Formulations during Storage and in Simulated Biological Fluid

The storage stability of the formulations was evaluated by monitoring vesicle mean diameter, PI, zeta potential, and ANP0903 content over two months at 4 ± 2 °C. Furthermore, since PEG-liposomes are intended for parenteral administration, their behavior in biological fluids was evaluated by incubating the formulations with Hank’s balanced salt solution (pH 7.4 ± 0.2), which was prepared according to the composition reported by Yang et al. (2007) [46]. The mean diameter, PI, and zeta potential of PEG-liposomes were measured immediately after dilution (1:10 *v*:*v*) of the formulations with Hank’s solution, and after 24 h of incubation at 37 °C.

### 4.4. Cell Culture and Treatments

Human hepatocellular carcinoma cell lines HepG2 and HuH7 were cultured in Dulbecco’s Modification of Eagle’s Medium (DMEM), supplemented with 10% fetal bovine serum, 100 IU/mL penicillin/streptomycin, and 2 mM L-glutamine. JHH6 cells, the third hepatocarcinoma cell line analyzed, were cultured in William’s E medium supplemented with 10% FBS, 2 mM L-glutamine, and 100 IU/mL penicillin/100 mg/mL streptomycin. The immortalized human hepatocytes (IHH) cell line, obtained through stable transfection, with the recombinant plasmid SV40, of healthy liver cells, was cultured in Dulbecco’s Modified Eagle Medium/Nutrient Mixture F-12 supplemented with 10% FBS, 2 mM L-glutamine, 100 IU/mL penicillin/streptomycin antibiotics, 1.2 × 10^−7^ M insulin from bovine pancreas, and 1 µM dexamethasone. The cells were grown in a humidified incubator with 5% CO_2_ at 37 °C. All experiments were performed using cells with less than 10 passages, in the logarithmic growth phase. HepG2 cells were purchased from ATCC (#HB-8065); HuH7 cell line was kindly provided by Prof. G. Giannelli (University of Bari, Italy). JHH6 and IHH cell lines were kindly donated by Prof. C. Tiribelli (Liver research Center, Italian Liver Foundation, Trieste, Italy).

ANP0903 synthetic compound was prepared as a 50 mM stock solution in dimethyl sulfoxide (DMSO). Drug concentrations were obtained by dilutions in complete growth medium so that the maximal final concentration of DMSO (0.8%) was well tolerated with no observable toxic effects to cells. In all experiments, cells treated with 0.8% DMSO were used as negative control.

### 4.5. MTT Assay

MTT (3-[4,5-dimethylthiazol-2-yl]-2,5-diphenyltetrazolium bromide) assay was performed to evaluate cytotoxic activities of both synthetic molecule ANP0903 in DMSO and ANP0903 PEG-liposomes (L-ANP0903). Empty PEG-liposomes (ELs) were used as a control. Briefly, cells were seeded in 96-well plates (1.5 × 10^4^ cells/well), treated with ANP0903 or L-ANP0903 at different concentrations (25, 50, 100, 200, and 400 µM), and incubated in a 5% CO_2_ incubator at 37 °C for 24 h. For comparative purposes, Els were tested at the same dilutions as those required for ANP0903 or L-ANP0903 to reach the above reported concentrations. The medium was then removed, 100 µL of MTT solution (0.75 mg/mL in complete DMEM) was added to each well, and the plate was incubated for 4 h in the dark at 37 °C in a cell culture incubator. The tetrazolium MTT was reduced in living cells in a mitochondrial-dependent reaction to insoluble purple formazan crystals, dissolved in a solution of isopropanol and DMSO (1:1). Cells treated with vehicle (DMSO) were used as a control. The absorbance was measured at 570 nm with background subtraction at 630 nm using a microplate reader (Multiskan Go spectrophotometer, Thermo Scientific). The half-maximal (50%) inhibitory concentration (IC_50_) was determined using GraphPad Prism software (version 8.4.2; GraphPad Software, La Jolla, CA, USA).

### 4.6. Morphological Analysis of Cells

HepG2 cells were cultured in 12-well plates (2 × 10^5^ cells per well), treated with ANP0903 in DMSO or ANP0903 PEG-liposomes at different concentrations (50 µM, 100 µM, and 200 µM) for 24 h and examined for morphological alterations using an inverted phase-contrast microscope (Nikon Eclipse TS100, Tokyo, Japan; 40× magnification). Cells treated with vehicle (DMSO) were used as a negative control.

### 4.7. Apoptosis Assay by Flow Cytometry

Apoptotic cell death, induced by treatment with ANP0903 in DMSO or liposomal ANP0903, was detected using Annexin V–FITC Apoptosis Detection Kit (BD Pharmingen, Franklin Lakes, NJ, USA) in accordance with the manufacturer’s instructions. Cells treated with vehicle (DMSO) were used as negative control. Briefly, cells were seeded in 24-well plates (1 × 10^5^ cells per well), cultured for 24 h, and exposed to different concentrations of free ANP0903 or liposomal ANP0903 (50, 100, and 200 µM) for 24 h at 37 °C. Subsequently, cells were harvested, washed twice with ice-cold PBS, resuspended in 100 µL of binding buffer, and stained with 5 µL of Annexin V–FITC and 5 µL of propidium iodide for 15 min in the dark at room temperature. An additional 400 μL of binding buffer was added to each tube and samples were analyzed by FACS Canto 37 II (excitation at 488 nm, emission at 585 nm) within 1 h.

### 4.8. Western Blot Analysis

HepG2 cells were grown in 6-well plates (5 × 10^5^ cells per well), exposed for 24 h at 37 °C to different concentrations of ANP0903, in DMSO solution or formulated in PEG-liposomes (50 µM, 100 µM and 200 µM), and finally lysed in RIPA buffer (Cell Signaling Technology CST, Danvers, MA, USA) supplemented with a protease inhibitor cocktail.

Protein concentration was determined using the Bradford assay [47]. An amount of 50 µg of each sample was loaded to SDS-PAGE gel electrophoresis and transferred onto nitrocellulose membranes. The membranes were incubated for 1 h at room temperature in blocking buffer (5% *w*/*v* non-fat dry milk in TRIS-buffered saline pH 7.4, supplemented with Tween 20 0.1% (*v*/*v*) (TBST)), followed by incubation with specific primary antibodies diluted in blocking buffer at 4 °C overnight (anti-BiP/GRP78 1:1000, anti-CHOP 1:1000, anti-IRE1α 1:1000, anti-sXBP1 1:1000, 1:1000, anti-PARP-1/cPARP-1 1:1000, anti-ubiquitin 1:1000, anti-Beclin-1 1:1000, anti-LC3 A/B 1:1000 anti-ATF6 1:1000, anti-p-PERK 1:1000, and anti-actin 1:1000).

Each membrane was then washed three times with TBST and incubated with the appropriate secondary antibody HRP-conjugated for 1 h at room temperature. After three washes with TBST, the membranes were exposed to enhanced chemiluminescence reagents (ECL Star Enhanced Chemiluminescent Substrate, LiteAblot TURBO Extra Sensitive Chemiluminescent Substrate, EuroClone, Milan, Italy) and the HRP signal was detected through the Chemidoc XRS detection system (BioRad, Hercules, CA, USA) with the ImageLab software (version 5.1; Bio-Rad, Hercules, CA, USA). Image J software (version 1.52a, National Institutes of Health, Bethesda, MD, USA) was used to process images.

### 4.9. LC-MS/MS Analysis

To quantify the cellular intake of ANP0903, HepG2 cells were seeded into 6-well plates at a density of 2.5 × 10^5^ cells/well, treated with 100 µM of free ANP0903 or liposomal ANP0903 for 3, 6, 12, and 24 h, and pelleted at 1200 rpm for 5 min. Supernatants were removed. To extract ANP0903, 500 µL of 40% MeOH with 0.1% (*v*/*v*) formic acid were added to the cells, which were lysed by sonication (1 min on/1 min off, 10 min total), mixed, and placed on ice for 15 min. Lysates were then centrifuged at 1500 rpm for 5 min. Supernatants were collected for LC–MS analysis. To ensure that all of the ANP0903 was extracted from cells, another amount of 250 µL of 40% MeOH with 0.1% (*v*/*v*) formic acid was added to the cell pellets and suspended again. After 15 min on ice, the suspensions were centrifuged at 14,000 rpm for 5 min, and all supernatants were pooled together [48,49,50]. The supernatants were dried with vacuum-centrifugation using a Concentrator plus (Eppendorf AG, Hamburg, Germany), the solid residues were dissolved in 200 µL of MeOH, and 10 µL aliquots were injected in an LC-ESI-Orbitrap Q Exactive system. The protein concentration in each sample was determined by the Bradford assay [47] and cell lysates were checked to verify success of the lyses step and to normalize multiple samples for side-by-side comparison. Our aim was to evaluate the amount of ANP0903 in differently treated cells. To this purpose, an Ultimate 3000 UPLC (Thermo Fisher Scientific, Milan, Italy) coupled with a high-resolution mass spectrometry Q Exactive Orbitrap (Thermo Fisher Scientific, Milan, Italy) was used. The selected chromatographic conditions were the following: Luna reverse phase C18 column (150 × 2 mm, 3 µm; Phenomenex, Torrance, CA, USA), using formic acid 0.1% (Eluent A) and acetonitrile (Eluent B) as mobile phase. A faster gradient from 30% to 100% of B in 15 min was used. The flow rate was 0.2 mL/min and the injection volume was 10 µL.

Full mass data were acquired in positive ion mode. MS analyses were performed by using product ion scan mode. Precursor ion at *m*/*z* 476.5, corresponding to the protonated ion of ANP0903 [M + H]^+^, was selected for the screening of ANP0903 in the samples of interest. The mass parameters were set up as follows: capillary temperature sets at 320 °C, flow rate of sheath gas and auxiliary gas set at 35.0, and 15 arbitrary units with a spray voltage of 3 KV. All the data were exported and processed with Xcalibur 2.2 SP1 software (Thermo Fisher Scientific, Milan, Italy) [51].

### 4.10. Statistical Analysis of Data

Results are presented as means ± Standard Errors (SE) or means ± Standard Deviations (SD). Shapiro–Wilk test was used to check if data follow a normal distribution. One-way analysis of variance (ANOVA) followed by Dunnett’s post hoc test or unpaired *t*-test were used to analyze normally distributed data. Nonparametric Kruskal–Walls or Mann–Whitney tests were chosen if the sample size was too small and did not fit the Gaussian distribution. All analyses were performed using GraphPad Prism software (version 8.4.2; GraphPad Software, La Jolla, CA, USA), considering *p*-values < 0.05 as statistically significant.

## 5. Conclusions

The proposed liposomal formulation was demonstrated to be an interesting tool for the incorporation of ANP0903. The PEG-liposomes were small in size, stable, and promoted the entry of the drug in hepatic cancer cells. The antitumor activity is probably due to proteasome inhibition. Further investigation will be needed to validate the in vitro potential of this formulation, for example in comparison with commercially available drugs.

## Figures and Tables

**Table 1:** Characteristics of empty and ANP0903 PEG-liposomes and non-PEGylated liposomes: mean diameter (MD), polydispersity index (PI), and zeta potential (ZP). Each value represents the mean ± SD (*n* > 10); * and ** values statistically different (*p* < 0.05 and *p* < 0.01, respectively) from empty PEG-liposomes and ANP0903 PEG-liposomes, respectively.

Formulation	MD nm ± SD	PI ± SD	ZP mV ± SD
Empty PEG-liposomes	87 ± 5.0	0.24 ± 0.03	−19 ± 6.5
ANP0903 PEG-liposomes	86 ± 6.2	0.24 ± 0.03	−17 ± 5.8
Empty liposomes	78 ± 4.8	0.30 ± 0.03	* −12 ± 2.7
ANP0903 liposomes	82 ± 1.9	0.34 ± 0.07	** −8 ± 2.3

**Table 2:** Mean diameter (MD), polydispersity index (PI), and zeta potential (ZP) of PEG-liposomes diluted and incubated with a simulated body fluid (Hank’s solution, pH 7.4) for 24 h, at 37 °C. The measurements were carried out immediately after the dilution (t_0_) and after 24 h (t_24_) of incubation. Mean values ± SD are reported (*n* = 6).

Formulation	Time	MD (nm ± SD)	PI ± SD	ZP (mV ± SD)
Empty PEG-liposomes	t_0_	85 ± 1.5	0.18 ± 0.01	−1.3 ± 0.5
	t_24_	80 ± 7.2	0.17 ± 0.01	−1.1 ± 0.2
ANP0903 PEG-liposomes	t_0_	83 ± 8.0	0.21 ± 0.05	−1.1 ± 0.3
	t_24_	80 ± 7.0	0.25 ± 0.08	−1.6 ± 0.6

**Table 3:** The half-maximal inhibitory concentration (IC_50_) values of ANP0903 and of its liposomal formulation (L-ANP0903) tested on human hepatocarcinoma cell lines. Empty PEG-liposomes (EL) were tested as a control to evaluate the cytotoxicity of the nanocarrier.

Cell Line	IC_50_ (µM)		
	ANP0903	L-ANP0903	EL
IHH	>400	>400	>400
JHH6	174	-	-
HuH7	161.7	-	-
HepG2	150.1	102.5	>400

**Table 4:** Composition of the liposomal formulations.

Formulation	DPPC	P90G	MPEG-5000-DPPE	ANP0903	EtOH	H_2_O
Empty PEG-liposomes	20 mg	20 mg	2 mg	--	50 µL	950 µL
ANP0903 PEG-liposomes	20 mg	20 mg	2 mg	2.4 mg (5 mM)	50 µL	950 µL
Empty liposomes	20 mg	20 mg	--	--	50 µL	950 µL
ANP0903 liposomes	20 mg	20 mg	--	2.4 mg (5 mM)	50 µL	950 µL

## Data Availability

The data presented in this study are available within this article.
